# Circular RNAs in Cardiovascular Diseases: From Regulatory Networks to Functional Effectors

**DOI:** 10.3390/ijms27125418

**Published:** 2026-06-16

**Authors:** Camilo Rebolledo, Luis A. Salazar

**Affiliations:** Centro de Biología Molecular y Farmacogenética, Departamento de Ciencias Básicas, Facultad de Medicina, Universidad de La Frontera, Temuco 4811230, Chile; camilo.rebolledo@ufrontera.cl

**Keywords:** circular RNAs, cardiovascular disease, cardiac remodeling, gene regulatory networks, fibrosis, hypertrophy, biomarkers, RNA therapeutics

## Abstract

Circular RNAs have emerged as important regulators of gene expression in cardiovascular disease, expanding the current understanding of the molecular mechanisms that underlie cardiac remodeling and dysfunction. Initially regarded as byproducts of aberrant splicing, circRNAs are now recognized as stable, abundant, and functionally versatile molecules with marked tissue specificity and diverse modes of action. In the cardiovascular system, circRNAs are generated through tightly regulated back-splicing mechanisms and act through multiple molecular pathways, including microRNA sequestration, protein scaffolding, modulation of transcription and splicing, regulation of mitochondrial and metabolic homeostasis, and in some cases, peptide translation. These properties position circRNAs as regulatory hubs that connect molecular interactions to functional cellular outcomes. Across a broad range of cardiovascular conditions, including heart failure, myocardial ischemia, fibrosis, arrhythmias, cardiotoxicity, and cardiorenal syndrome, circRNAs have been implicated in processes such as hypertrophy, inflammation, cell death, extracellular matrix remodeling, and regenerative responses. Beyond their mechanistic relevance, circRNAs also hold preclinical relevance as circulating biomarkers and therapeutic targets owing to their stability in biofluids and their capacity to modulate disease-relevant networks. Nevertheless, major challenges remain, including incomplete functional validation, methodological heterogeneity, annotation inconsistencies, and barriers to clinical translation. In this review, we synthesize the current knowledge on circRNA biogenesis, molecular function, disease-specific roles, biomarker potential, and therapeutic applications and discuss the conceptual and technical advances required to move the field from descriptive association toward mechanistic and clinical impact.

## 1. Introduction

Cardiovascular diseases (CVDs) remain the leading cause of morbidity and mortality worldwide, encompassing a wide spectrum of pathological conditions including myocardial infarction, hypertension, metabolic disorders, and systemic inflammation, all of which converge on progressive cardiac dysfunction and adverse remodeling [1,2,3]. Despite major advances in clinical management, disease progression remains insufficiently controlled in many patients, reflecting persistent gaps in our understanding of the molecular processes that drive cardiovascular pathology.

At the core of cardiovascular disease lies a complex and dynamic reprogramming of gene expression, involving coordinated regulation at transcriptional, post-transcriptional, and epigenetic levels [4,5]. In this context, non-coding RNAs (ncRNAs) have emerged as critical regulators of cardiovascular biology. RNA-mediated regulation operates within interconnected molecular networks that integrate multiple RNA species and protein interactions [6,7].

Circular RNAs (circRNAs), generated through back-splicing events, have gained increasing attention as key components of this regulatory landscape [8,9]. These molecules are abundant, evolutionarily conserved, and exhibit marked tissue specificity, particularly in the heart [10,11]. Once considered splicing byproducts, circRNAs are now recognized as stable and functionally versatile molecules capable of interacting with microRNAs, RNA-binding proteins, and the translational machinery [12,13].

A predominant framework for circRNA function has been their role as microRNA sponges, whereby they sequester miRNAs through microRNA response elements (MREs) and modulate downstream gene expression [14,15]. While this model has provided important mechanistic insights, it represents only a partial view of circRNA biology. Emerging evidence suggests that circRNAs operate within complex, condition-specific regulatory networks, engaging in RNA–RNA, RNA–protein, and protein–protein interactions that collectively shape cellular response profiles [16,17]. In addition, circRNAs can influence transcriptional regulation, act as protein scaffolds, and in some cases, encode functional peptides, expanding their role beyond canonical post-transcriptional control [18,19].

In cardiovascular disease, numerous circRNAs have been identified as differentially expressed and functionally relevant regulators of pathological processes such as fibrosis, hypertrophy, inflammation, and cardiomyocyte survival [20,21,22]. However, these findings remain largely fragmented, often centered on individual molecular interactions or isolated regulatory axes without integrating them into broader frameworks or linking them to downstream functional outcomes. This reductionist perspective limits our ability to fully understand how circRNA-driven regulatory circuits contribute to cardiovascular remodeling and disease progression at a systems level.

In this review, we propose a framework in which circRNAs are viewed not as isolated regulators, but as dynamic nodes within gene expression networks that translate molecular interactions into functional cellular outcomes. To develop this perspective, we first examine the mechanisms that underlie circRNA biogenesis and regulation in the cardiovascular system. We subsequently examine their principal molecular functions, relate these functional roles to disease-specific pathological contexts, and discuss their emerging relevance as circulating biomarkers and therapeutic targets. Finally, we consider the main challenges and future directions that will define the clinical and mechanistic maturation of the field.

## 2. Materials and Methods

This study was conducted as a narrative review aimed at providing an integrative and concept-driven synthesis of the role of circular RNAs (circRNAs) in cardiovascular disease. A structured literature search was performed using the PubMed, Scopus, and Web of Science databases to identify relevant publications addressing circRNA biology in cardiovascular contexts. The search strategy combined controlled vocabulary and free-text terms, including “circRNA”, “circular RNA”, “cardiovascular disease”, “heart failure”, “ceRNA network”, and “biomarker”. Additional records were identified through the manual screening of reference lists from selected articles. The literature search primarily focused on studies published between 2015 and 2025, while earlier landmark studies were included when conceptually relevant.

Studies were considered eligible if they addressed mechanistic, diagnostic, prognostic, or therapeutic aspects of circRNAs in cardiovascular diseases. Priority was given to peer-reviewed original research articles, recent high-quality reviews, and studies with translational relevance, including those incorporating human clinical data, independent validation cohorts, or mechanistic experimental evidence. In cases of overlapping literature, preference was given to more recent, comprehensive, or methodologically robust publications. Particular attention was paid to studies reporting experimental platform, biospecimen type, cohort characteristics, sample size, and validation strategies. The selected literature was synthesized using a narrative and thematic approach. Evidence was organized into major conceptual domains, including circRNA biogenesis and regulation, molecular mechanisms of action, disease-specific roles, biomarker potential, and therapeutic applications. This approach enabled integration across multiple levels of biological organization, from molecular interactions to functional and clinical outcomes.

Given the narrative nature of this review, no formal systematic review protocol or quantitative meta-analysis was applied. Likewise, no standardized risk-of-bias assessment tool was used across all studies. Instead, the included literature was critically appraised using qualitative criteria relevant to translational cardiovascular research, including study design, experimental model, sample size, biospecimen definition, methodological rigor, mechanistic validation, reproducibility across independent datasets, and clinical relevance. Evidence derived from multicohort human studies, independent validation analyses, or convergent findings across experimental systems was considered stronger than evidence based on small exploratory cohorts or isolated preclinical observations.

## 3. Biogenesis and Regulatory Properties of circRNAs in the Cardiovascular System

To understand how circRNAs shape cardiovascular phenotypes, it is first necessary to examine the mechanisms that determine their generation, regulation, and molecular behavior within the cardiovascular system. These upstream features provide the basis for the diverse biological functions explored in subsequent sections.

Circular RNAs (circRNAs) are generated through a non-canonical splicing event known as back-splicing, in which a downstream 5′ splice donor is covalently joined to an upstream 3′ splice acceptor. This process results in the formation of covalently closed RNA molecules lacking both a 5′ cap and a 3′ polyadenylated tail, conferring resistance to exonuclease-mediated degradation and contributing to their enhanced stability relative to linear RNAs [8,9]. In the cardiovascular system, circRNAs exhibit abundant and tightly regulated expression patterns, supporting the view that their biogenesis represents a controlled and biologically meaningful process rather than a byproduct of splicing noise [10,11].

### 3.1. Back-Splicing Mechanisms and circRNA Diversity

CircRNA biogenesis results from a coordinated interplay between cis-regulatory elements and trans-acting factors that determine back-splicing efficiency and shape the circRNA landscape. Unlike canonical linear splicing, back-splicing involves the covalent linkage of a downstream splice donor to an upstream splice acceptor, a process that is intrinsically less favorable and therefore tightly regulated [23,24]. This mechanism introduces an additional layer of transcriptome complexity, allowing a single genomic locus to generate both linear and circular RNA isoforms with distinct structural and functional properties.

At the cis-regulatory level, intronic complementary sequences play a central role in facilitating circularization. In particular, inverted repeat elements such as Alu sequences promote the formation of RNA duplexes that bring splice sites into close spatial proximity, thereby enhancing back-splicing efficiency [23,25]. The presence, orientation, and distance between these elements critically influence circRNA output, underscoring genomic architecture as a key determinant of circRNA biogenesis [24,25]. Additional mechanisms, including exon skipping and lariat intermediate formation, further expand the diversity of circularization pathways [10,26].

RNA-binding proteins represent key regulators of circRNA formation by modulating splice site selection. Proteins such as Muscleblind-like splicing regulator 1 (MBNL1) and Quaking (QKI) promote circularization by binding flanking intronic regions and stabilizing RNA secondary structures required for back-splicing [27,28]. Importantly, these RBPs display tissue-specific expression patterns and regulatory dynamics, providing a mechanistic basis for the cell-type specificity of circRNAs [11,29]. In contrast, other splicing factors favor canonical exon joining, establishing a competitive balance between linear and circular RNA processing.

RNA editing adds another level of regulation. Adenosine-to-inosine (A-to-I) editing mediated by adenosine deaminases acting on RNA (ADAR) enzymes disrupts intronic base pairing within double-stranded RNA regions, thereby reducing back-splicing efficiency [25]. In cardiovascular tissues, altered ADAR activity has been linked to changes in circRNA abundance under pathological conditions, including heart failure [30], highlighting the responsiveness of circRNA biogenesis to disease-associated regulatory shifts.

Transcriptional dynamics also contribute to circRNA production. Variations in RNA polymerase II elongation rates influence splice site recognition and the temporal window for back-splicing, thereby modulating the balance between linear and circular isoforms [24]. This co-transcriptional dimension is particularly relevant in the cardiovascular system, where transcriptional programs respond rapidly to mechanical stress, metabolic signals, and injury.

Transcriptomic analyses have revealed extensive circRNA diversity in the heart across cardiomyocytes, fibroblasts, and endothelial cells [10,11]. Notably, circRNA expression often does not correlate with that of their linear host genes, indicating that back-splicing is independently regulated and contributes selectively to gene regulatory outputs [26,29]. Together, these mechanisms generate diverse classes of circRNAs, including exonic circRNAs (ecircRNAs), exon–intron circRNAs (EIciRNAs), and circular intronic RNAs (ciRNAs), each with distinct structural and functional properties [31,32].

### 3.2. Regulation of circRNA Formation

**Figure 1:**
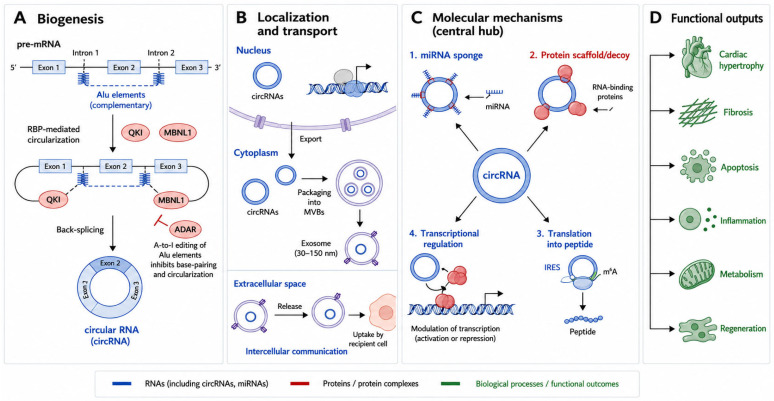
Biogenesis, localization, and molecular functions of circRNAs in cardiovascular disease. (**A**) circRNAs arise from precursor mRNAs through back-splicing, regulated by intronic complementary sequences, RBPs such as QKI and MBNL1, and RNA-editing enzymes including ADAR. (**B**) After biogenesis, circRNAs localize to nuclear and cytoplasmic compartments or are packaged into extracellular vesicles, supporting intercellular communication. (**C**) Functionally, circRNAs act as regulatory nodes by modulating microRNA activity through MREs, interacting with proteins as scaffolds or decoys, regulating transcription, and in selected cases, encoding peptides via IRES- or m6A-dependent translation. (**D**) These activities influence hypertrophy, fibrosis, apoptosis, inflammation, metabolic adaptation, and regeneration, linking circRNA-mediated regulation to cardiovascular remodeling. Figure created by the authors using Canva v2026.05 (Canva Pty Ltd., Sydney, Australia) with royalty-free graphical elements.

While back-splicing provides a structural basis for circRNA generation, the abundance and disease-associated behavior of circRNAs depend on dynamic regulation of this process. CircRNA formation is shaped by the coordinated activity of RBPs, splicing factors, and RNA editing enzymes that together control splice site selection and back-splicing efficiency (Figure 1).

RBPs such as QKI and MBNL1 enhance circRNA production by stabilizing RNA secondary structures and facilitating splice site proximity [27,28], thereby enabling the context-dependent generation of circular RNA isoforms. In contrast, ADAR-mediated RNA editing acts as a negative regulator of circRNA biogenesis. By modifying double-stranded RNA regions within intronic complementary sequences, ADAR enzymes disrupt base pairing and limit circularization [9,25]. In human cardiac tissue, reduced ADAR2 activity has been associated with increased circRNA abundance, directly linking RNA editing to disease-associated circRNA dysregulation [30].

CircRNA production is also shaped by competition with canonical splicing. Because back-splicing and linear splicing share splice sites, changes in spliceosome activity, exon inclusion, or intron retention could shift the balance between transcript isoforms [33,34]. In the cardiovascular system, where splicing programs are highly responsive to developmental and pathological cues, circRNA generation is therefore embedded within broader regulatory networks controlling gene expression [35].

Under pathological conditions such as pressure overload, ischemia, or inflammation, alterations in RBPs, splicing regulators, and RNA editing enzymes lead to widespread remodeling of circRNA expression profiles [10,11,30]. Emerging studies indicate that disease-relevant circRNAs are frequently produced under conditions of perturbed RNA processing, linking upstream regulatory mechanisms to downstream functional effects. For instance, circRNAs such as circSMAD4 and circMap4k2 are induced under stress conditions associated with remodeling and regeneration, reinforcing the connection between circRNA biogenesis and disease-specific regulatory programs [20,21].

### 3.3. Structural Features Associated with circRNA Function

The same regulatory features that govern circRNA production also shape their biological activity. In particular, stability, tissue specificity, and subcellular localization define how circRNAs accumulate, where they operate, and which molecular partners they engage.

#### 3.3.1. Stability

A defining feature of circRNAs is their exceptional stability, resulting from their covalently closed structure, which lacks free 5′ and 3′ ends and confers resistance to exonucleolytic degradation. This stability allows circRNAs to accumulate, particularly in long-lived cells such as cardiomyocytes, where they may exert sustained regulatory effects [10,26].

Despite this stability, circRNAs are not inert. Mechanisms including endonuclease-mediated cleavage and N6-methyladenosine (m6A)-dependent pathways contribute to circRNA degradation, linking their turnover to broader epitranscriptomic regulation [36,37]. In addition, circRNAs could be selectively packaged into extracellular vesicles and released into circulation, representing an active export mechanism that also supports intercellular communication [38,39].

#### 3.3.2. Tissue Specificity

CircRNAs exhibit pronounced tissue and cell-type specificity, particularly in the cardiovascular system. Distinct expression profiles have been identified across cardiomyocytes, fibroblasts, and endothelial cells, reflecting their specialized roles in cardiac homeostasis [8,10]. Notably, circRNA abundance does not necessarily correlate with that of their linear host genes, indicating independent regulatory control.

CircRNA expression is also developmentally regulated, with specific subsets enriched in fetal versus adult hearts, suggesting roles in cardiac maturation and differentiation [8]. Under pathological conditions, including myocardial infarction and cardiomyopathies, circRNA expression undergoes extensive remodeling and is associated with processes such as fibrosis, hypertrophy, and inflammation [20,21,40]. Many circRNAs therefore display cell-type-specific expression patterns that support localized regulatory responses within the cardiac microenvironment.

#### 3.3.3. Subcellular Localization

The subcellular localization of circRNAs is closely linked to their functional roles. Exonic circRNAs are predominantly cytoplasmic, where they interact with miRNAs and RBPs to regulate post-transcriptional gene expression [9]. By contrast, exon–intron circRNAs and circular intronic RNAs are often retained in the nucleus, where they could modulate transcriptional processes, including regulation of parental gene expression [36].

In cardiomyocytes, this spatial organization influences functional outcomes. Cytoplasmic circRNAs participate in pathways controlling hypertrophy, apoptosis, and inflammation, whereas nuclear circRNAs may contribute to longer-term transcriptional remodeling. In parallel, extracellular vesicle-associated circRNAs enable intercellular communication within the cardiac microenvironment, particularly between endothelial cells, fibroblasts, and cardiomyocytes [15,38] (Figure 1).

## 4. Molecular Mechanisms of circRNA Function in Cardiovascular Biology

The regulatory features that define circRNA biogenesis and distribution provide the foundation for their diverse molecular functions. These functions extend well beyond transcript abundance and position circRNAs as active participants in cardiovascular gene regulatory networks.

CircRNAs exert their biological functions through a diverse repertoire of molecular mechanisms that extend beyond the classical competing endogenous RNA (ceRNA) mechanisms. circRNAs participate in regulatory networks by interacting with microRNAs (miRNAs), RNA-binding proteins, and, in some cases, the translational machinery. These interactions allow circRNAs to couple molecular signals with coordinated cellular responses, shaping key processes involved in cardiovascular physiology and disease [8,9,17]. The diverse molecular mechanisms through which circRNAs regulate gene expression and cellular processes are summarized in Table 1.

Importantly, the mechanisms summarized in Table 1 should not be interpreted as isolated functional categories, rather, they represent complementary regulatory layers through which circRNAs connect RNA-level control with protein activity, transcriptional regulation, organelle homeostasis, and peptide-mediated signaling. This view is particularly relevant in cardiovascular disease, where remodeling emerges from the convergence of multiple molecular perturbations rather than from a single dominant regulatory axis [18,32,41,42,43].

### 4.1. circRNA–miRNA Interactions: Beyond the Sponge Mechanism

One of the earliest and most widely explored functions attributed to circRNAs is their ability to interact with miRNAs. In cardiovascular biology, this concept initially gained traction through the canonical competing endogenous RNA framework, in which circRNAs act as molecular sponges that sequester miRNAs and relieve the repression of downstream mRNA targets [14,45]. Foundational studies demonstrated that this mechanism could exert biologically relevant effects, particularly when circRNAs are sufficiently abundant and interact with key regulatory miRNAs [14,45].

However, this model does not fully capture the complexity of circRNA–miRNA interactions. Many circRNAs contain limited numbers of microRNA response elements, and their regulatory effects depend strongly on cellular context, transcript abundance, and subcellular localization. Rather than acting as simple stoichiometric inhibitors, circRNAs often reshape signaling programs by modulating the timing, threshold, and robustness of gene expression responses [16,45,46].

Hypertrophy. CircHRCR protects against pathological hypertrophy by sponging miR-223 and preserving ARC expression. Conversely, circCacna1c promotes hypertrophy via miR-29b-2-5p/NFATc1, while circNfix attenuates hypertrophy through miR-145-5p/ATF3 [47,48]. These interactions illustrate how circRNAs could either amplify or restrain stress-responsive transcriptional programs depending on network context.

Fibrosis. CircRNA-mediated miRNA regulation also controls fibroblast activation and extracellular matrix deposition. circHIPK3 promotes fibrosis via miR-29b-3p, while circSMAD4 regulates fibroblast activation through miR-671-5p/FGFR2 signaling [20,49]. These interactions operate at the level of coordinated signaling pathways, including TGF-β and Wnt.

Apoptosis and survival. CircRNAs regulate cardiomyocyte survival under stress. Cdr1as aggravates myocardial infarction via miR-7a, whereas circSNRK protects through miR-103-3p/GSK3β signaling [50,51]. Similar axes operate in diabetic cardiomyopathy and cardiotoxicity, indicating that circRNAs modulate thresholds for cell death across multiple pathological contexts [52,53].

Inflammation. CircRNA–miRNA interactions also regulate inflammatory signaling. circ_0003907 promotes inflammation via miR-944 and the MYD88/NLRP3/NF-κB axis, linking circRNAs to innate immune activation and myocardial injury [54].

Despite this mechanistic relevance, ceRNA-like activity accounts for only part of what cardiovascular circRNAs do. The sections that follow address protein scaffolding, transcriptional modulation, mitochondrial regulation, and peptide translation, all of which operates independently of miRNA sequestration, and taken together, paints a considerably richer picture of circRNA biology (Figure 1C) [19,32,41,42,44].

### 4.2. circRNA–Protein Interactions: Scaffolding and Regulatory Complexes

In addition to RNA-level regulation, circRNAs could directly interact with proteins. In these contexts, circRNAs act as scaffolds, adaptors, or decoys, shaping signaling outputs by recruiting proteins, stabilizing complexes, or modulating protein activity [8,12]. Cardiovascular examples such as circITGa9 and circSMAD3 illustrate how circRNA–protein interactions can influence cytoskeletal organization and profibrotic signaling pathways [41,42].

These examples show that circRNA–protein interactions can directly influence structural and signaling programs involved in pathological remodeling. In the case of circITGa9, binding to TPM3 provides a mechanism through which a circRNA can modulate actin polymerization and fibroblast-associated cytoskeletal remodeling, thereby linking RNA regulation to extracellular matrix remodeling and fibrosis [41]. Similarly, circSMAD3 illustrates how circRNAs can act as protein decoys or scaffolds within canonical profibrotic signaling pathways, since recruitment of YBX1 reduces SMAD3 phosphorylation and attenuates TGF-β/SMAD-dependent cardiac remodeling [42]. These findings support the view that circRNAs can modulate protein complex behavior and signaling output independently of miRNA sequestration.

CircRNAs also regulate metabolic and mitochondrial pathways. FEACR stabilizes NAMPT, sustaining a cardioprotective metabolic axis [55]. circSamd4 localizes to mitochondria and interacts with valosin-containing protein (VCP), a key regulator of mitochondrial protein quality control, thereby reducing ROS production and promoting cardiac repair [44]. Mitochondrial circRNAs (mecciRNAs) further expand this landscape. These mecciRNAs have been shown to interact with proteins involved in mitochondrial protein import and homeostasis, thereby linking circRNA biology to mitochondrial function and stress adaptation [43]. CircRYR2 has been linked to calcium handling through interactions with calcium-regulatory protein complexes, suggesting that circRNAs could directly influence excitation–contraction coupling [56].

The metabolic and mitochondrial roles of circRNAs further demonstrate the functional diversity of non-ceRNA mechanisms. FEACR interacts with NAMPT and suppresses ferroptosis during myocardial ischemia/reperfusion injury, connecting circRNA–protein interaction with metabolic resilience and cardiomyocyte survival [55]. circSamd4 provides another example of organelle-associated regulation, as its interaction with VCP limits mitochondria-derived ROS and promotes post-infarction cardiac repair [44]. In addition, mecciRNAs have been implicated in mitochondrial homeostasis and explored as exogenous therapeutic molecules in heart failure, reinforcing the concept that circRNAs can operate within organelle-specific regulatory systems rather than exclusively within cytoplasmic miRNA networks [43].

Nuclear circRNAs, particularly EIciRNAs and ciRNAs, can also engage transcriptional machinery and modulate parental gene expression, providing a non-ceRNA route through which circRNAs contribute to longer-term transcriptional remodeling [12]. This mechanism is less well-characterized than cytoplasmic protein interactions but is particularly relevant to chronic cardiovascular disease progression, where durable changes in gene-expression programs dominate [32].

### 4.3. Protein-Coding Potential of circRNAs

The long-standing view of circRNAs as non-coding molecules has been revised by the discovery that a subset can be translated into functional peptides [57,58]. This adds a new dimension to circRNA biology, positioning them as both regulatory RNAs and protein-coding entities [9,13].

CircRNA translation is generally mediated by cap-independent processes, including internal ribosome entry site (IRES)-driven translation and m6A-dependent initiation, with particular relevance under stress conditions such as ischemia or hypertrophy. CircCDYL encodes the peptide tCDYL-100, which promotes hypertrophy, while circNlgn produces a protein that drives fibrosis via nuclear signaling pathways [18,19]. These examples demonstrate that circRNA-derived peptides could actively modulate cardiac remodeling.

The functional consequences go beyond simply producing an additional protein product. circCDYL encodes tCDYL-100, a peptide that exacerbates cardiac hypertrophy by disrupting the REST–CDYL–EHMT2 transcriptional repression complex and activating pro-hypertrophic gene programs [18]. circNlgn produces Nlgn173, which drives fibroblast proliferation and collagen deposition through LaminB1-associated nuclear signaling pathways [19]. In cardiac fibroblasts, circ_0036176-derived Myo9a-208 modulates fibroblast proliferation with apparent anti-fibrotic effects, illustrating that circRNA-encoded peptides can exert either pathogenic or counter-regulatory roles depending on context [59]. Landmark studies demonstrating IRES- and m6A-mediated circRNA translation, including circZNF609 in myogenesis, established the conceptual framework for viewing circRNAs as dual-function molecules [57,58]. Representative examples are summarized in Table 2.

### 4.4. circRNAs as Regulators of Core Cellular Processes

More broadly, circRNA-derived proteins may act as signaling intermediates, transcriptional modulators, or structural regulators, thereby expanding the functional repertoire of circRNAs beyond RNA-based regulation. At this level, the distinction between coding and non-coding circRNA functions becomes functionally blurred. A single circRNA may contribute to cellular remodeling through RNA–RNA interactions, protein binding, organelle-associated regulation, or peptide production. In our view, these regulatory processes influence core cellular programs that determine whether cardiac cells adapt to stress, engage in maladaptive remodeling, or progress toward irreversible injury [18,19,44,55,59].

The molecular interactions described above converge on a limited set of cellular processes that are central to cardiovascular pathology. In cell death, circRNAs control apoptosis, ferroptosis, and pyroptosis. circCmiss1 promotes ferroptosis via EIF4A3/TfR1, whereas circ-0006332 enhances pyroptosis through miR-143/TLR2 [60,61]. In inflammation, circRNAs regulate immune signaling pathways, including MYD88/NLRP3/NF-κB, linking inflammatory activation to myocardial injury [54]. In metabolism and mitochondria, circRNAs influence metabolic pathways and mitochondrial function, including lipid metabolism, ROS production, and fibroblast energy homeostasis [62]. In regeneration, circMap4k2 and circWhsc1 promote cardiomyocyte proliferation and repair through defined signaling axes, highlighting their role in regenerative responses [15,21].

### 4.5. circRNA-Centered Regulatory Networks

These mechanistic layers are best understood not as isolated events, but as components of broader molecular architectures. Within these systems, circRNAs can connect miRNAs, mRNAs, proteins, and disease-associated signaling pathways, thereby linking molecular regulation to functional remodeling in cardiovascular disease [16,17]. The circRNA–miRNA–mRNA axes represent one of the best-characterized network structures, but increasing evidence indicates that circRNAs operate within higher-order regulatory systems rather than simple linear pathways [10,63].

Multi-omics approaches (integrating RNA-Seq, proteomics, pathway enrichment, and co-expression modeling) have enabled the reconstruction of circRNA-centered networks and the identification of regulatory hubs [26,64]. Within this framework, circRNAs do not simply regulate single targets; they modulate local network topology by influencing miRNA availability, protein complex organization, transcriptional output, mitochondrial adaptation, and peptide-mediated signaling in concert. The aggregate effect is that circRNAs translate distributed regulatory perturbations into coordinated cellular responses affecting fibrosis, hypertrophy, inflammation, metabolic adaptation, regeneration, and disease progression simultaneously [16,17,40,65].

## 5. circRNAs Across Cardiovascular Diseases: Integrating Mechanisms with Pathology

The mechanistic frameworks described above are especially informative when examined in the context of disease, where circRNA-mediated interactions converge on pathological processes such as hypertrophy, fibrosis, inflammation, and cardiomyocyte injury. The functional relevance of circRNAs in cardiovascular biology therefore becomes particularly evident when their molecular actions are linked to specific pathological settings.

**Figure 2:**
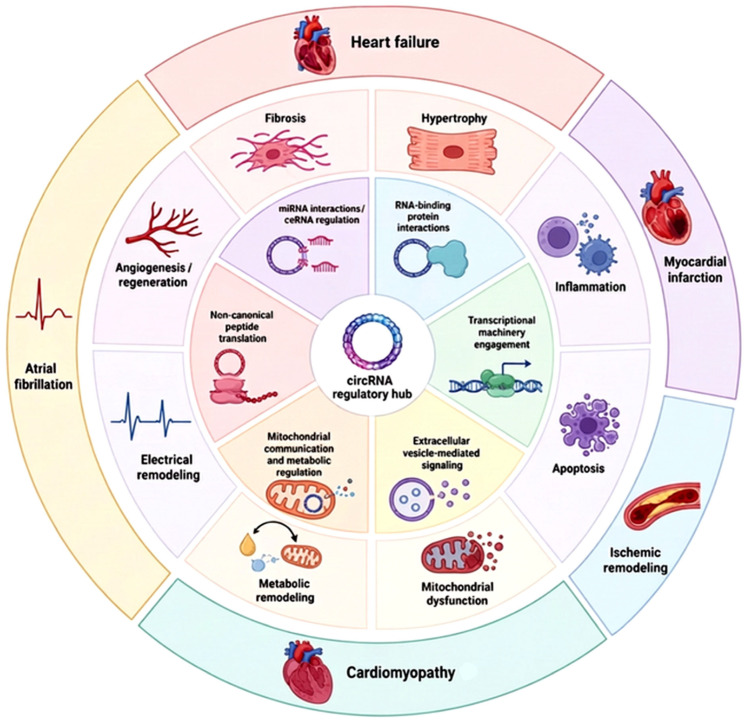
CircRNAs as integrative regulators of cardiovascular remodeling. CircRNAs participate in cardiovascular disease through multilayer regulatory interactions that extend beyond canonical miRNA sequestration. Through interactions with miRNAs, RNA-binding proteins (RBPs), transcriptional machinery, extracellular vesicle-mediated signaling pathways, mitochondrial communication systems, and non-canonical peptide translation mechanisms, circRNAs contribute to the regulation of interconnected cellular remodeling programs. These include fibrosis, hypertrophy, inflammation, apoptosis, mitochondrial dysfunction, metabolic remodeling, electrical remodeling, and angiogenesis/regeneration. The integration of these regulatory networks ultimately influences the development and progression of major cardiovascular phenotypes, including heart failure, myocardial infarction, ischemic remodeling, cardiomyopathy, and atrial fibrillation. Figure created by the authors using Canva v2026.05 (Canva Pty Ltd., Sydney, Australia) with royalty-free graphical elements.

circRNAs participate in interconnected regulatory networks that converge on core pathological processes, including hypertrophy, fibrosis, inflammation, metabolic dysregulation, and cell death. Across cardiovascular diseases, circRNAs exhibit context-dependent expression patterns, reflecting their capacity to link molecular interactions with phenotypic outcomes that drive cardiac remodeling and dysfunction [8,17,20]. These integrated mechanisms and their relationship to major cardiovascular phenotypes are summarized in Figure 2.

This disease-oriented perspective is essential because the functional consequence of a circRNA-mediated mechanism may vary according to cell type, disease stage, and tissue context. Thus, the relevance of a given circRNA is not determined solely by its direct molecular target, but by how its regulatory activity influences broader remodeling programs such as fibroblast activation, cardiomyocyte stress responses, mitochondrial adaptation, inflammatory signaling, and tissue repair [8,10,17,41].

### 5.1. Cardiac Remodeling and Heart Failure

Cardiac remodeling encompasses structural, cellular, and molecular adaptations in response to hemodynamic stress, injury, or neurohormonal activation. These include cardiomyocyte hypertrophy, fibroblast proliferation, extracellular matrix remodeling, mitochondrial dysfunction, and inflammatory signaling. Several circRNAs have been implicated in these processes, suggesting that circRNA-mediated regulation may contribute to multiple layers of gene expression control [8,13,41,65].

CircRNAs regulate hypertrophic responses through circRNA–miRNA–mRNA axes and protein interactions that modulate signaling pathways such as PI3K/AKT, MAPK, and calcineurin–NFAT. circHRCR protects against pathological hypertrophy via the miR-223/ARC axis, whereas circCacna1c promotes hypertrophy through miR-29b-2-5p/NFATc1 signaling [14,47]. In contrast, circMap4k2 enhances cardiomyocyte proliferation and alleviates residual remodeling following post-myocardial infarction surgical ventricular restoration, linking regenerative responses with hypertrophic signaling [21].

Cardiac fibrosis is a central component of adverse remodeling and a major determinant of ventricular stiffness and dysfunction, characterized by fibroblast activation, myofibroblast differentiation, and excessive extracellular matrix deposition [66,67]. A central regulatory axis involves TGF-β/Smad signaling, in which TGF-β activation induces the phosphorylation of Smad2/3, nuclear translocation, and the transcription of profibrotic genes. CircRNAs modulate this pathway at multiple levels. For example, circSMAD4 promotes myofibroblast activation via the miR-671-5p/FGFR2 axis, linking circRNA regulation to canonical profibrotic signaling [20]. Other circRNAs, including circHIPK3 and circ_000203, regulate extracellular matrix gene expression through antifibrotic miRNA sequestration, reinforcing fibroblast activation and collagen deposition [49,68].

These examples suggest that circRNAs participate in heart failure not as isolated regulators of individual targets, but as modulators of interconnected remodeling programs. Hypertrophy-associated circRNAs influence stress-responsive transcriptional circuits involving calcium-dependent and NFAT-associated signaling, whereas fibrosis-associated circRNAs converge on fibroblast activation, extracellular matrix deposition, and TGF-β/Smad-related pathways [14,20,47,49,68]. At the same time, regenerative circRNAs such as circMap4k2 and circWhsc1 link post-injury repair with proliferative and survival signaling [15,21]. Thus, circRNA-mediated regulation in heart failure is better interpreted as a multilayered process linking cardiomyocyte growth, fibroblast activation, mitochondrial stress, inflammation, and regenerative capacity to the transition from compensated remodeling to ventricular dysfunction [17,69].

Representative circRNAs involved in cardiovascular remodeling and disease processes, together with their mechanisms of action and functional effects, are summarized in Table 3. The combined effects of hypertrophy, fibrosis, metabolic dysregulation, and inflammation drive progressive functional decline, thereby contributing to the transition from compensated remodeling to heart failure [17,69].

### 5.2. Myocardial Ischemia and Ischemia–Reperfusion Injury

Myocardial ischemia and subsequent reperfusion trigger complex pathological responses involving cardiomyocyte death, oxidative stress, inflammation, and adverse remodeling. CircRNAs function as key regulators of these processes by integrating stress-responsive signaling networks [12,55,65].

CircRNAs modulate multiple forms of regulated cell death, including apoptosis, pyroptosis, and ferroptosis. For instance, circ_0001312 regulates apoptosis and oxidative stress via the miR-409-3p/HMGB1 axis, while circWhsc1 enhances cardiomyocyte survival and proliferation under hypoxic conditions through TRIM59/STAT3/Cyclin B2 signaling [15,52]. In parallel, circRNAs such as circCmiss1 link iron metabolism to ferroptosis, highlighting their role in redox homeostasis and post-ischemic remodeling [60].

Beyond intracellular regulation, circRNAs participate in intercellular communication through extracellular vesicles (EVs), acting as signaling mediators within the cardiac microenvironment. EV-associated circRNAs could modulate fibroblast activation, endothelial responses, and immune cell polarization, thereby coordinating multicellular responses during cardiac injury and repair [70,71]. This mechanism extends circRNA function beyond cell-autonomous regulation, highlighting their role in tissue-level remodeling dynamics.

The convergence of ferroptosis, pyroptosis, and mitochondrial ROS dysregulation during ischemia–reperfusion positions circRNAs as regulators at the intersection of redox and cell death signaling, a role distinct from simple miRNA buffering. This integrated view is important because ischemia–reperfusion injury reflects the convergence of mitochondrial dysfunction, regulated cell death, immune activation, and maladaptive tissue remodeling rather than a single molecular lesion. From this perspective, circRNAs may serve as regulatory intermediates that bridge intracellular stress responses with multicellular reparative or injury-amplifying programs [15,51,60,70,71].

### 5.3. Pressure Overload-Induced Cardiac Hypertrophy

CircRNAs regulate hypertrophic growth through the coordinated control of transcriptional, post-transcriptional, metabolic, and stress-response pathways. These mechanisms converge on key transcription factors such as NFAT, GATA4, and MEF2, integrating mechanical and biochemical stimuli [14,56].

Thus, circRNAs could act as either pro- or anti-hypertrophic regulators. circHRCR inhibits hypertrophy via miR-223, whereas circCacna1c and circLarp4b promote hypertrophic signaling through the NFAT and MEF2 pathways, respectively [14,72]. Additional regulatory layers include autophagy and ferroptosis, with circSIRT1 promoting protective autophagy and circCmiss1 linking iron metabolism to hypertrophic stress responses [60,73].

Furthermore, circRNA-derived peptides, such as tCDYL-100, demonstrate that circRNAs could directly modulate chromatin-associated transcriptional programs, extending their regulatory capacity beyond RNA-mediated mechanisms [18].

This mechanistic diversity is instructive. Hypertrophy-associated circRNAs collectively influence calcium-dependent transcriptional activation, stress-responsive gene expression, autophagy, ferroptosis, and peptide-mediated transcriptional regulation [14,18,56,60,72,73]. Whether a given circRNA buffers or amplifies pressure overload-induced remodeling depends on its molecular partners and the cellular state, context-dependency that cannot be resolved by profiling expression alone.

### 5.4. Cardiac Fibrosis and Extracellular Matrix Remodeling

CircRNAs regulate fibrotic remodeling through the coordinated control of fibroblast activation, extracellular matrix production, cytoskeletal organization, and metabolic adaptation. Beyond canonical ceRNA activity, circRNAs such as circITGa9 directly interact with structural proteins, including TPM3, thereby modulating cytoskeletal dynamics and fibroblast transformation [41].

They also play a role in modulating fibrosis through metabolic pathways. For example, circIGF1R modulates glycolytic reprogramming in fibroblasts, linking metabolic shifts to fibrotic activation [62]. In addition, extracellular circRNAs contribute to intercellular signaling within the cardiac microenvironment, coordinating fibroblast–cardiomyocyte interactions through vesicle-mediated communication [15].

Fibrotic remodeling therefore provides one of the clearest examples of how circRNAs integrate multiple regulatory layers. At the post-transcriptional level, circRNAs such as circSMAD4, circHIPK3, and circ_000203 regulate miRNA-dependent control of profibrotic gene expression and extracellular matrix deposition [20,49,68]. At the protein level, circITGa9 links circRNA function to cytoskeletal remodeling through TPM3-dependent actin polymerization [41]. At the metabolic and intercellular levels, circRNAs influence fibroblast energy remodeling and extracellular vesicle-mediated communication within the cardiac microenvironment [15,62]. These regulatory pathways collectively promote fibroblast activation, matrix deposition, tissue stiffening, and the gradual deterioration of ventricular function.

### 5.5. Arrhythmias and Atrial Fibrillation

Atrial arrhythmogenesis depends on coordinated changes in conduction velocity, refractoriness, calcium handling, and tissue architecture, all of which are susceptible to regulation by circRNA networks [10,74]. CircRNAs appear to contribute primarily through two well-documented mechanisms: structural remodeling and inflammatory signaling.

Regarding structural remodeling, circHIPK3-driven fibroblast activation promotes atrial fibrosis, a key substrate for reentry, and circRNA signatures associated with atrial fibrosis have been reported in patient samples [49,75]. Regarding inflammation, circRNAs embedded within NF-κB-associated regulatory networks contribute to the immune-mediated tissue vulnerability that sustains electrical instability [76]. A third, less validated possibility is direct electrophysiological modulation: candidate circRNAs linked to calcium-handling proteins and ion-channel pathways have been reported, but mechanistic evidence remains sparse, and this relationship deserves dedicated investigation [10,77].

From a mechanistic perspective, atrial fibrillation should be interpreted as a disease context in which circRNAs may connect structural and electrical remodeling. Fibrosis-associated circRNAs can contribute to conduction heterogeneity by promoting extracellular matrix deposition, whereas inflammation-associated circRNA networks may amplify atrial tissue vulnerability and electrical instability [49,75,76]. In parallel, candidate circRNAs linked to calcium handling and ion-channel-associated pathways suggest a possible direct contribution to electrophysiological remodeling, although this remains less functionally validated than the roles of circRNAs in fibrosis or inflammation [10,77]. This distinction is important because it separates well-supported remodeling mechanisms from more exploratory electrophysiological hypotheses.

### 5.6. Other Cardiovascular Conditions

CircRNAs also play roles in additional cardiovascular contexts, including cardiotoxicity, sepsis-related cardiac injury, and cardiorenal syndrome, where they integrate inflammatory, metabolic, and stress-response pathways.

In cardiotoxicity, circRNAs can either exacerbate or mitigate injury depending on context. circ_0001312 promotes apoptosis and oxidative stress, whereas circITCH and circINSR exert protective effects by restoring metabolic and survival pathways [52,78]. In sepsis-related cardiac injury, circRNAs amplify inflammatory signaling through pathways such as MYD88/NLRP3/NF-κB, contributing to oxidative stress and myocardial dysfunction [54]. Transcriptome-wide analyses further reveal extensive circRNA dysregulation associated with immune activation, suggesting that circRNAs are embedded in systemic inflammatory remodeling [79]. In cardiorenal syndrome, circRNA–miRNA–mRNA networks coordinate cross-organ communication between the heart and kidney, with the shared dysregulation of pathways such as PI3K/AKT linking fibrosis, hypertrophy, and metabolic dysfunction [63,80].

Although these conditions differ clinically, they share common remodeling logic: circRNAs appear to participate in the coordination of stress responses that include inflammation, oxidative injury, metabolic imbalance, apoptosis, fibrosis, and organ crosstalk. In cardiotoxicity, this regulation is centered on survival and oxidative stress pathways [52,78,81]; in sepsis-related cardiac injury, it is dominated by inflammatory amplification [54,79]; and in cardiorenal syndrome, it involves shared heart–kidney regulatory networks linking fibrosis, hypertrophy, and metabolic dysfunction [63,80]. These examples further support the view that circRNAs operate across diverse cardiovascular stress states in a specific manner, rather than as disease-specific molecules with fixed biological roles.

## 6. circRNAs as Circulating Biomarkers in Cardiovascular Disease

The disease-associated expression patterns and functional relevance of circRNAs have also stimulated interest in their use as circulating biomarkers capable of reflecting ongoing pathological remodeling. The identification of reliable biomarkers for cardiovascular diseases remains a major clinical challenge, especially for the early diagnosis, risk stratification, and monitoring of therapeutic responses. circRNAs have shown potential in preliminary studies due to their intrinsic stability, tissue specificity, and detectability in body fluids. Their presence in circulation reflects both passive release from damaged cells and active secretion via extracellular vesicles, positioning circRNAs as informative indicators of underlying pathological processes [8,12,26].

### 6.1. Detection in Plasma, Serum, and Extracellular Vesicles

The detection of circRNAs in plasma, serum, and extracellular vesicles has become a central element in evaluating their translational relevance in cardiovascular disease. A key advantage of circRNAs as circulating analytes is their structural stability, which contributes to their persistence in extracellular environments and facilitates reliable detection across a range of experimental conditions [10,26].

CircRNAs can be detected in biofluids using quantitative reverse transcription PCR, microarray platforms, and high-throughput RNA sequencing. qRT-PCR remains the standard for targeted validation due to its sensitivity and specificity, whereas RNA sequencing enables the unbiased identification of novel circRNAs with potential clinical relevance [26,69]. Methodological studies have demonstrated that RNA extraction protocols, library preparation strategies, and normalization procedures significantly influence analytical reproducibility, highlighting the need for standardized workflows when profiling circulating RNAs [82,83,84,85].

A substantial fraction of circulating circRNAs is associated with extracellular vesicles, including exosomes and microvesicles. This vesicular packaging not only protects circRNAs from degradation but also facilitates their transfer between cells, suggesting a dual role as both biomarkers and mediators of intercellular communication [38,70]. Evidence from studies on exosomal circRNAs indicates their involvement in both pathogenic and reparative signaling processes across cardiovascular conditions, including ischemic injury and heart failure, thereby reinforcing their functional relevance beyond passive release mechanisms [38].

Pre-analytical and analytical variables remain critical determinants of circRNA detection. Factors such as sample source, hemolysis, centrifugation protocols, vesicle isolation methods, and the presence of protein- or lipoprotein-associated RNAs could affect RNA yield and composition. These variables are particularly relevant given the relatively low abundance of many circulating circRNAs, where technical variability may obscure biologically meaningful signals if not carefully controlled [82,83].

### 6.2. Diagnostic and Prognostic Potential

Circulating circRNAs have shown emerging potential as diagnostic and prognostic biomarkers for cardiovascular diseases. Their relative stability and tissue-associated expression profiles support non-invasive assessment of cardiac pathology while reflecting underlying molecular processes such as fibrosis, inflammation, and remodeling [26,70,82], although substantial technical and clinical validation challenges remain.

Multiple studies have reported differential circRNA expression across cardiovascular conditions, including heart failure, myocardial infarction, and atrial fibrillation. These disease-associated circRNAs have shown promising sensitivity and specificity in selected studies, supporting their potential utility in clinical diagnostics [20,21,86].

In heart failure, circulating circRNAs such as circCDR1as, circBPTF, and circPRDM5 correlate with ventricular dysfunction, fibrosis, and disease severity, underscoring their prognostic relevance [70]. Similarly, in ischemic heart disease, circRNAs are dynamically regulated following myocardial infarction and have been associated with infarct size and clinical outcomes, suggesting value for both early diagnosis and disease monitoring [26,69].

In atrial fibrillation, specific circRNA signatures have demonstrated diagnostic potential, including the ability to distinguish new-onset disease with high sensitivity and specificity [77]. These findings are consistent with broader network analyses showing that circRNAs are embedded within regulatory circuits governing fibrosis, inflammation, and electrical remodeling.

An additional strength of circRNAs lies in their integration into multi-marker strategies. Combining circRNA profiles with traditional biomarkers and clinical parameters may improve predictive accuracy and support more refined patient stratification, consistent with precision medicine approaches [70]. Importantly, most circRNA biomarker studies remain exploratory and are frequently limited by small cohorts, heterogeneous analytical methodologies, and lack of independent multicenter validation, which currently restricts their direct clinical applicability.

Representative circRNA signatures associated with distinct cardiovascular phenotypes and remodeling programs are summarized in Table 4. Beyond simple disease association, these circRNAs illustrate how circRNA profiling could contribute to precision-oriented cardiovascular research, including risk stratification, disease monitoring, therapeutic response assessment, and the identification of patient-specific remodeling trajectories.

### 6.3. Limitations and Challenges in Clinical Translation

Despite their promise, several challenges limit the clinical implementation of circRNA-based biomarkers. A primary limitation is the lack of standardized protocols for circRNA detection and quantification. Variability in pre-analytical steps, including sample collection, processing, storage, RNA extraction, and library preparation, could influence circRNA profiles and lead to inconsistencies across studies [26,82]. Differences in sequencing platforms, bioinformatic pipelines, and normalization strategies further complicate reproducibility and cross-study comparisons [12,16].

A second challenge is the relatively low abundance of many circRNAs in circulation, particularly in early disease stages. Although their stability provides an advantage, sensitive and optimized detection methods are still required to ensure reliable quantification [9,26].

Biological variability also represents a major limitation. CircRNA expression can be influenced by age, sex, comorbidities, medication use, and lifestyle factors, complicating interpretation and limiting generalizability [69]. In addition, intra-individual variability and potential circadian effects remain poorly characterized [70].

Another critical issue is the incomplete understanding of the biological origin and functional relevance of circulating circRNAs. Distinguishing between actively secreted circRNAs and those released as a consequence of tissue damage remains challenging, which complicates their interpretation as mechanistic biomarkers [9,17].

Finally, most studies to date rely on relatively small cohorts, limiting statistical power and reproducibility. Large-scale, multicenter studies and prospective validation are necessary to establish the clinical utility of circRNAs and their added value relative to established biomarkers [26,77].

## 7. Therapeutic Potential of circRNA-Targeted Strategies

**Figure 3:**
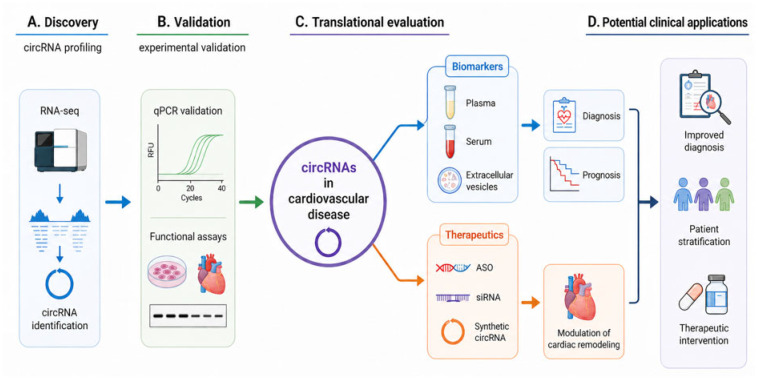
Translational pipeline of circRNA research in cardiovascular disease: from discovery to potential clinical application. Progression of circRNA research from molecular discovery to translational evaluation in cardiovascular disease. (**A**) CircRNAs are initially identified through high-throughput profiling approaches, such as RNA sequencing, (**B**) and subsequently validated using targeted methods including quantitative PCR and functional assays. (**C**) As integrative regulators of gene expression networks, circRNAs provide a mechanistic bridge between molecular alterations and disease phenotypes. In the clinical context, circulating circRNAs detected in plasma, serum, and extracellular vesicles represent minimally invasive biomarker candidates for diagnosis and prognosis. In parallel, experimental therapeutic strategies targeting circRNAs, including antisense oligonucleotides directed against back-splice junctions, RNA interference approaches, and engineered synthetic circRNAs, enable the preclinical modulation of pathways involved in cardiac remodeling. (**D**) These complementary biomarker and therapeutic applications may contribute to improved diagnostic accuracy, patient stratification, and personalized therapeutic development if current barriers in validation, delivery, safety, and clinical reproducibility are systematically addressed. Figure created by the authors using Canva v2026.05 (Canva Pty Ltd., Sydney, Australia) with royalty-free graphical elements.

Beyond their value as measurable indicators of disease, circRNAs are increasingly being explored as potential therapeutic targets, and in some cases, as therapeutic molecules in their own right. The growing recognition of circRNAs as regulators of gene expression networks in cardiovascular disease has opened experimental avenues for intervention, particularly in preclinical models of cardiac remodeling, fibrosis, injury, and regeneration. Unlike traditional approaches that target single genes or proteins, circRNA-based strategies may, in principle, allow for the modulation of selected disease-relevant regulatory modules, thereby influencing multiple interconnected pathways simultaneously. This is in line with the emerging understanding that circRNAs function as key coordinators of molecular interactions that drive cardiac remodeling and dysfunction [8,16,17]. However, this same network-level activity also raises important concerns regarding specificity, dosage control, and unintended perturbation of compensatory regulatory circuits. The transition from molecular discovery to therapeutic application is schematically illustrated in Figure 3.

Accordingly, the therapeutic potential of circRNAs should be interpreted as an emerging and still largely preclinical field. While individual studies demonstrate that circRNA silencing, replacement, or extracellular vesicle-mediated delivery can modify disease-relevant phenotypes, the clinical maturity of these approaches remains substantially lower than that of other RNA-based therapeutic platforms. Lessons from cardiovascular RNA therapeutics, including antisense- and microRNA-directed strategies, highlight that target engagement, tissue delivery, immune activation, and long-term safety remain central barriers even for more established RNA modalities [88,89,90].

### 7.1. circRNAs Within the Landscape of RNA-Based Cardiovascular Therapies

RNA-based therapies have gained traction as strategies to modulate disease-relevant pathways in cardiovascular disease. Initial approaches have largely targeted microRNAs or mRNA-related mechanisms, but their broad and pleiotropic activity has raised concerns regarding specificity and unintended off-target effects [91]. This has driven interest toward alternative RNA classes, including circRNAs, which exhibit enhanced stability and network-level regulatory capacity.

Nevertheless, the therapeutic development of circRNA-based strategies should be positioned within the broader challenges of RNA medicine. In cardiovascular disease, RNA therapeutics must overcome barriers related to cardiac tissue delivery, endosomal escape, cell-type specificity, durability of effect, dose titration, and immune compatibility. These issues are not unique to circRNAs, but they may be amplified by the fact that circRNAs can interact with multiple molecular partners and participate in broader regulatory networks [13,88].

CircRNAs offer distinct molecular features, including enhanced stability, cell-type specificity, and the ability to engage multiple molecular partners simultaneously. These features position them as attractive but still insufficiently validated candidates for next-generation RNA-based therapies [9,12]. Recent studies have demonstrated that circRNAs can be therapeutically manipulated either by silencing pathogenic molecules or by delivering synthetic circRNAs with beneficial functions [92,93]. These approaches enable the modulation of key signaling pathways involved in cardiac remodeling, regeneration, and stress responses in preclinical models.

Advances in delivery systems, including viral vectors, lipid nanoparticles, and extracellular vesicles, have further supported the translational potential of RNA-based therapies, particularly by enabling tissue-specific targeting within the heart [38,94]. Although circRNA-based approaches show considerable experimental potential, multiple barriers remain before clinical translation becomes feasible, including delivery specificity, off-target effects, long-term safety, immunogenicity, scalability, and disease stage-dependent regulatory complexity.

### 7.2. Targeting circRNAs

Targeting circRNAs represents a theoretically specific therapeutic strategy enabled by their unique back-splice junction, which allows for selective targeting without affecting linear transcripts. Antisense oligonucleotides designed against the BSJ could induce circRNA degradation or functional inhibition with high specificity [20,95]. However, this specificity must be experimentally verified, since circRNAs may share sequence features with host transcripts, alternative isoforms, or related circular products from the same genomic locus. Current therapeutic strategies targeting circRNAs, including their mechanisms and experimental applications, are summarized in Table 5.

Preclinical studies demonstrated that circRNA silencing could modulate disease-relevant pathways. For example, the inhibition of circ_0001312 reduces cardiotoxicity by attenuating apoptosis and oxidative stress, whereas targeting circSMAD4 suppresses fibrosis through disruption of the miR-671-5p/FGFR2 axis [20,52]. CircRNA targeting also affects broader regulatory networks, classical examples include circHRCR, which modulates hypertrophy via miR-223, and circMap4k2 and circWhsc1, which influence proliferation, fibrosis, and repair following myocardial injury [14,15,21].

These examples provide valuable proof-of-concept evidence; however, they should not be construed as demonstrating immediate or near-term clinical translatability. Most circRNA-targeting studies remain confined to cell culture systems or animal models, and their therapeutic relevance depends on reproducible target engagement, durable phenotypic benefit, and the absence of adverse effects in clinically meaningful settings. For example, circSMAD4 targeting provides a fibrosis-focused model, circ_0001312 silencing illustrates protection in doxorubicin-induced cardiotoxicity, and EV-mediated circWhsc1 delivery supports regenerative repair after myocardial injury; however, each of these strategies still requires validation across dose, timing, delivery route, and disease stage [15,20,52].

In addition to silencing, modulation of circRNA biogenesis through RNA-binding proteins such as QKI, MBNL1, and ADAR represents an emerging strategy to reshape circRNA expression landscapes [9,12]. Overexpression or replacement strategies further expand the therapeutic scope. Delivery of protective circRNAs, such as EV-mediated circWhsc1, could enhance cardiomyocyte proliferation and improve cardiac repair, thereby demonstrating the feasibility of restoring beneficial regulatory circuits [15].

However, targeting circRNA biogenesis may be less specific than targeting individual circRNAs. RBPs and RNA editing enzymes control many linear and circular transcripts simultaneously, so interventions aimed at QKI, MBNL1, ADAR, or related processing factors could produce broad transcriptomic effects. Such approaches may be useful experimentally, but therapeutic translation would require the careful separation of beneficial circRNA remodeling from unintended effects on canonical splicing, RNA editing, or host-gene expression.

Overall, achieving efficient delivery, molecular specificity, appropriate dosage, and long-term control of circRNA modulation remains a key translational challenge.

### 7.3. circRNAs as Therapeutic Molecules

Beyond serving as targets, circRNAs could function as therapeutic molecules themselves. Their covalently closed structure provides enhanced stability and prolonged activity compared with linear RNAs, making them conceptually attractive for sustained therapeutic applications [9,13]. These same properties require careful control over persistence, intracellular localization, translation potential, and degradation.

Engineered circRNAs could be designed to perform diverse functions: as miRNA sponges incorporating optimized MREs to sequester disease-promoting miRNAs; as protein decoys or scaffolds that modulate signaling pathways through protein interactions; or as templates for peptide translation, enabling the sustained production of therapeutic proteins [9,13]. Nevertheless, the design of engineered circRNAs must consider immunogenicity, unintended protein production, saturation of endogenous RNA-binding proteins or miRNAs, and the possibility of disrupting physiological regulatory networks.

Preclinical studies provide proof-of-concept, circWhsc1 enhances cardiomyocyte proliferation and cardiac repair via TRIM59/STAT3 signaling, while circMap4k2 promotes regenerative responses through miR-106a-3p/Azin1 modulation [15,21]. CircRNAs also modulate fibrosis and cell survival. For instance, circ_0001312 targeting reduces cardiotoxicity, whereas circSorbs1 has been associated with enhanced regeneration and reduced apoptosis [52,96].

The therapeutic use of circRNAs as molecules rather than targets is therefore promising but technically demanding. EV-mediated circWhsc1 delivery illustrates how circRNAs may be transferred to injured myocardium in a reparative context, whereas circMap4k2 and circSorbs1 highlight the potential of circRNA-associated regenerative signaling after injury or cardiotoxic stress [15,21,96]. However, these examples remain preclinical, and future studies must determine whether engineered or delivered circRNAs can achieve reproducible cardiac distribution, controlled duration of activity, and safety across heterogeneous cardiovascular disease settings.

Delivery platforms, including lipid nanoparticles and extracellular vesicles, may enable functional circRNA transfer, although optimizing tissue specificity, scalability, and safety remains a major challenge [71,97].

Extracellular vesicles are particularly relevant because they can function as both natural carriers of regulatory RNAs and as experimental delivery vehicles, but their therapeutic use is still limited by heterogeneity in vesicle source, isolation method, cargo loading, biodistribution, and potency assessment [71]. Similarly, lipid nanoparticle-based circRNA delivery is an active area of development, but the optimization of formulation, immune response, and tissue targeting remains necessary before extrapolation to cardiovascular therapy [97].

### 7.4. Interaction with Conventional Therapies

CircRNA-based interventions are likely to complement rather than replace existing therapies. Their role as regulators of gene expression networks positions them to influence therapeutic efficacy, responsiveness, and adverse effects [8,98]. This concept remains largely exploratory and requires stronger evidence linking circRNA modulation to clinically meaningful treatment response.

CircRNAs could interact bidirectionally with conventional treatments, and their modulation may complement existing therapies by targeting overlapping pathways and reshaping key signaling networks [17,69]. On the other hand, pharmacological treatments could alter the circRNA expression profiles, thereby indirectly influencing regulatory networks. This interaction is exemplified by statins, which improve endothelial function partly through the upregulation of circRBCK1 and modulation of the GTPCH1/eNOS pathway [99]. Similarly, metabolic therapies have been proposed to exert part of their effects through the modulation of ncRNA networks, including circRNAs [13,100].

This bidirectional relationship suggests that circRNA profiles may eventually contribute to therapy stratification or pharmacodynamic monitoring. For example, circRBCK1 links statin-associated endothelial protection with nitric oxide signaling, whereas transcriptomic analyses in patients treated with sacubitril/valsartan after acute myocardial infarction suggest that circRNA-centered networks may reflect therapeutic heterogeneity and remodeling trajectories [99,101]. However, these findings should be interpreted as hypothesis-generating until validated in prospective and clinically stratified cohorts.

CircRNAs may also serve as adjuncts in cardiotoxicity. For example, circ_0001312 reduces doxorubicin-induced cardiac injury by modulating apoptosis and inflammation pathways [52]. In regenerative contexts, circRNAs enhance therapeutic outcomes. circMap4k2 supports myocardial repair following surgical ventricular restoration, while EV-delivered circWhsc1 promotes cardiomyocyte proliferation, suggesting synergy with cell-based therapies [15,21]. Although circRNAs may contribute to therapy stratification through their association with disease severity and remodeling status, their primary value lies in their ability to integrate and modulate multiple therapeutic pathways simultaneously [70].

Despite this potential, adjunctive circRNA-based therapy will require careful evaluation of timing and disease context. A circRNA that is beneficial during acute repair may be ineffective or harmful during chronic remodeling if it amplifies proliferation, fibrosis, or maladaptive stress signaling. Therefore, therapeutic use of circRNAs should be guided by disease stage, target cell type, molecular mechanism, and expected duration of action.

Overall, circRNAs represent a promising, but still insufficiently validated, interface between molecular regulation and clinical intervention. Their therapeutic value will depend on whether the coordinated modulation of complex disease networks can be achieved with sufficient specificity, reproducibility, and safety in clinically relevant cardiovascular settings.

## 8. Current Challenges and Future Directions

Despite these opportunities, the path toward clinical implementation remains constrained by several conceptual, technical, and translational challenges that continue to shape the field. Many of these limitations arise from the intrinsic complexity of circRNA biology, particularly their integration within regulatory networks that govern cardiac remodeling and disease progression [8,16,80]. These considerations call for a more critical interpretation of the field. Although thousands of circRNAs have been cataloged across cardiovascular tissues, disease states, and circulating compartments, only a small fraction has been functionally characterized in sufficient depth [10,11,65]. For example, large-scale studies have described extensive circRNA repertoires in human, mouse, and rat hearts, whereas mechanistic validation has generally focused on a limited number of candidates such as circHRCR, circSMAD4, circWhsc1, circMap4k2, circITGa9, and circSamd4. Moreover, many proposed circRNA functions remain inferred from differential expression, predicted binding relationships, or isolated gain- and loss-of-function assays. This creates a persistent gap between circRNA discovery and mechanistic validation, particularly when assigning causal roles in complex cardiovascular phenotypes [10,13,16].

### 8.1. Functional Validation Gaps

A major bottleneck in the field is the discrepancy between the large number of identified circRNAs and the relatively small subset that has been functionally validated. High-throughput studies have cataloged thousands of circRNAs across tissues and disease states, yet only a limited number have been investigated beyond differential expression or predicted interactions [10,26].

Functional characterization often relies on reductionist approaches such as overexpression or knockdown of single circRNAs in vitro, without adequately considering their integration into broader regulatory networks. This is particularly problematic because circRNA function is highly context dependent and influenced by cell type, disease stage, subcellular localization, and molecular environment [8,16]. A circRNA that appears strongly pro-remodeling in one cellular context may exert neutral, compensatory, or even protective effects in another, reflecting the network-dependent nature of its activity. For instance, circRNAs implicated in fibrosis, regeneration, or mitochondrial regulation may exert different functional consequences depending on whether they operate in fibroblasts, cardiomyocytes, endothelial cells, extracellular vesicles, or organelle-associated compartments [15,20,44].

This context dependency also helps explain why apparently conflicting findings may emerge across studies. Differences in experimental model, disease stage, cellular composition, perturbation strategy, and analytical pipeline can substantially alter the observed functional effect of a given circRNA. For example, circRNA signatures identified in transverse aortic constriction models may reflect stage-specific remodeling programs, whereas human end-stage heart failure samples capture a more advanced and clinically heterogeneous disease state [102,103]. Therefore, inconsistent results should not always be interpreted as experimental disagreement alone; in some cases, they may reflect genuine biological differences in how circRNA-mediated regulation operates across cellular states and disease contexts.

Another limitation is that many functional studies still focus on predicted circRNA–miRNA–mRNA axes without fully testing whether the proposed interaction is quantitatively plausible or biologically dominant. Effective ceRNA-like activity depends on circRNA abundance, subcellular localization, microRNA availability, and competition with other target transcripts. As a result, some reported axes may represent partial or context-specific regulatory effects rather than major drivers of gene expression. This issue is particularly relevant for low-abundance circRNAs detected only through high-throughput sequencing or computational prediction, where functional relevance requires additional validation by targeted quantification, perturbation assays, and downstream phenotypic analysis [10,45,102,104].

Additionally, much of the available evidence derives from animal models or simplified systems, thereby limiting translational relevance. Differences in circRNA conservation, back-splice junction sequences, and cardiac physiology across species further complicate extrapolation to human disease [14,15,21]. Addressing these limitations will require the increased use of human tissues, iPSC-derived systems, and clinically annotated cohorts [69,105].

Species differences are particularly relevant for circRNAs because their back-splice junctions, flanking intronic sequences, expression patterns, and cellular abundance may not be fully conserved between experimental models and humans. Comparative analyses have shown both conserved and species-specific circRNA expression patterns across human, mouse, and rat hearts, underscoring the need for caution when extrapolating preclinical findings to human cardiovascular disease [10,11]. Consequently, a circRNA with robust functional effects in a mouse model may not have a direct human ortholog, may differ in abundance, or may participate in a distinct regulatory network in human cardiovascular tissue. This limitation is especially important for therapeutic target selection, where the conservation of sequence, expression context, and disease relevance must be established before translational conclusions can be drawn [40,69].

### 8.2. Reproducibility and Heterogeneity

Reproducibility remains a major challenge in circRNA research. Variability in sample processing, RNA extraction, and sequencing platforms could lead to inconsistent results across studies [26,82]. Additionally, biological heterogeneity, including differences in cell-type composition and patient characteristics, complicates the interpretation of circRNA profiles [69,70].

Low circRNA abundance represents an additional source of analytical and biological uncertainty. Although circRNAs are structurally stable, many disease-associated circRNAs are expressed at low levels, especially in plasma, serum, or extracellular vesicle preparations. This complicates detection, normalization, and cross-study comparison, and may also limit the likelihood that some circRNAs exert strong stoichiometric effects on miRNA availability under physiological conditions. Methodological variables, including RNA isolation, RNase R treatment, library preparation, sequencing depth, divergent primer design, and qPCR normalization, can further influence whether low-abundance circRNAs are reproducibly detected [26,84,106].

CircRNA expression is highly cell-type-specific and dynamically regulated, and bulk analyses may obscure differences between cardiomyocytes, fibroblasts, endothelial cells, and immune populations. Patient-level variables, including age, sex, comorbidities, and treatment status, add another layer of variability, limiting the identification of robust biomarkers or therapeutic targets [69,70].

These sources of heterogeneity are especially problematic in cardiovascular disease, where tissue samples often contain changing proportions of cardiomyocytes, fibroblasts, endothelial cells, vascular cells, and infiltrating immune populations. Consequently, changes in bulk circRNA abundance may reflect altered cell composition rather than true regulation within a specific cell type. For example, circRNA expression profiles obtained from whole cardiac tissue may obscure whether a candidate circRNA originates predominantly from cardiomyocytes, fibroblasts, immune cells, or extracellular vesicle-associated compartments [10,76,107]. Future studies should therefore prioritize cell-type-aware approaches, including purified cell populations, iPSC-derived cardiovascular models, spatially resolved analyses, or integration with cell-type deconvolution frameworks [40,69].

### 8.3. Standardization of circRNA Annotation

A major barrier to cumulative progress is the lack of standardized circRNA annotation and nomenclature. Different studies frequently report circRNAs using inconsistent identifiers, genomic coordinates, or naming conventions, which complicates cross-study comparisons and meta-analyses [8,10]. Annotation inconsistency also affects functional interpretation; a circRNA described under different names, genomic coordinates, or database identifiers may be difficult to compare across studies, even when it originates from the same host gene. Conversely, circRNAs derived from the same locus may differ in exon composition, back-splice junction sequence, or subcellular localization, making it inappropriate to assume functional equivalence based solely on host-gene identity. This issue is particularly relevant when comparing mechanistic studies, biomarker reports, and computational network reconstructions [34,108,109].

This problem is exacerbated by variability in computational pipelines for circRNA detection. Differences in back-splice junction identification, read filtering, and quantification strategies could lead to substantial discrepancies in reported circRNA repertoires [26]. The absence of unified standards limits reproducibility and hampers integration with other omics datasets.

Establishing consensus guidelines for circRNA annotation, together with standardized bioinformatic workflows and publicly accessible databases, will be essential to improve data interoperability and accelerate translational progress.

### 8.4. Translational Barriers

Although circRNAs hold strong promise as biomarkers and therapeutic targets, their translation into clinical practice remains constrained by multiple factors. For biomarker applications, key challenges include technical variability, limited multicenter validation, low circulating abundance, and incomplete understanding of the biological origin of circulating circRNAs [15,26,84]. For example, circulating circRNAs may originate from damaged cardiomyocytes, activated fibroblasts, endothelial cells, immune cells, or extracellular vesicle-mediated release, making it difficult to determine whether they reflect tissue injury, active intercellular signaling, or systemic remodeling. These limitations indicate that most circRNA biomarker candidates remain exploratory and that major barriers must be addressed before routine clinical use becomes feasible.

For therapeutic applications, major obstacles include efficient and tissue-specific delivery, off-target effects, dosage control, immunogenicity, and long-term safety. Importantly, circRNAs are embedded within complex regulatory networks and may share sequence features with host transcripts or related isoforms. Although targeting the back-splice junction improves specificity, broader consequences must be carefully anticipated and experimentally evaluated because perturbing a single circRNA may affect multiple downstream pathways, cellular processes, or compensatory regulatory circuits. This is relevant for strategies involving ASOs, RNA interference, synthetic circRNAs, extracellular vesicle delivery, or the manipulation of circRNA biogenesis, all of which remain largely preclinical in cardiovascular disease [13,15,20,43].

A further challenge is the temporal dimension of circRNA regulation. Most available studies analyze circRNA expression at a single disease stage or experimental time point, whereas cardiovascular remodeling is a dynamic process involving early injury responses, compensatory adaptation, chronic maladaptation, and terminal dysfunction. Stage-specific analyses in transverse aortic constriction models and transcriptomic studies in end-stage human heart failure illustrate that circRNA-associated networks may differ substantially across disease progression and etiology [102,103]. Without longitudinal sampling, it remains difficult to distinguish circRNAs that initiate disease progression from those that reflect secondary adaptation, tissue damage, or late-stage remodeling.

### 8.5. Integration with Multi-Omics and AI Approaches

Future progress will depend on integrating circRNA research within multi-omics and systems biology frameworks. CircRNAs interact with miRNAs, proteins, metabolites, and, in some cases, the translational machinery, requiring integrative approaches that combine transcriptomics, proteomics, epigenomics, and spatial transcriptomics to reconstruct disease-relevant regulatory networks [64,69].

Artificial intelligence and machine learning tools further enhance this integrative capacity by enabling the prioritization of candidate circRNAs based on network connectivity, conservation, and clinical association [26,110]. Benchmarking studies of computational methods for circRNA–disease association prediction highlight both the potential of these approaches and their dependence on data quality, annotation consistency, and experimental validation [109]. Therefore, computational prioritization should be interpreted as a hypothesis-generating strategy rather than definitive evidence of functional relevance.

A particularly promising direction is the integration of multi-omics datasets with perturbation-based functional genomics. Approaches combining CRISPR-based screens, BSJ-targeting ASOs, ribosome profiling, proteomics, and spatial transcriptomics will be critical to move the field from correlative associations toward mechanistic understanding and predictive modeling of cardiovascular disease [16,64].

In this context, future studies should aim to link circRNA expression with measurable functional outputs, including pathway activation, protein complex remodeling, mitochondrial function, extracellular matrix deposition, electrophysiological properties, and clinical outcomes. Examples such as circSMAD4 in fibrosis, circITGa9 in cytoskeletal remodeling, circSamd4 in mitochondrial ROS regulation, and circWhsc1 in post-infarction repair illustrate how circRNA function can be strengthened when molecular interactions are connected to cellular and tissue-level phenotypes [15,20,41,44].

### 8.6. Future Perspectives

Challenges in circRNA research reflect a field that has expanded rapidly in descriptive scope but now requires consolidation through rigorous validation, standardization, and integrative analysis. Functional validation gaps, reproducibility issues, inconsistent annotation, and translational barriers currently limit the confidence with which circRNAs could be positioned as clinical biomarkers or therapeutic targets [8,13,65].

At the same time, advances in human tissue resources, iPSC-derived cardiovascular models, multi-omics technologies, spatially resolved analysis, and AI-driven prioritization provide an opportunity to overcome these limitations. Future progress will depend on combining high-quality human-centered studies with standardized analytical frameworks and mechanistically informed experimental models [40,69,109].

If these challenges are systematically addressed, circRNAs may eventually transition from emerging regulators of gene expression to clinically actionable mediators of diagnosis, prognosis, and therapy in cardiovascular disease. However, this transition will require moving beyond differential expression and predicted interactions toward quantitative, cell-type-resolved, longitudinal, and functionally validated models of circRNA biology [13,16,69].

## 9. Conclusions

Circular RNAs have emerged as functionally relevant regulators of cardiovascular biology, expanding the current understanding of gene regulation in cardiac remodeling and disease progression. The evidence reviewed herein supports a conceptual framework in which circRNAs are best understood not merely as microRNA sponge molecules, but as dynamic regulatory nodes that coordinate multiple layers of gene regulation, including RNA–RNA interactions, RNA–protein binding, transcriptional modulation, mitochondrial and metabolic control, and in selected biological contexts, peptide translation [8,9,13,18,19].

This broader mechanistic perspective is relevant in cardiovascular disease, where pathological remodeling results from the convergence of hypertrophy, fibrosis, inflammation, cell death, mitochondrial dysfunction, metabolic adaptation, electrical remodeling, and impaired repair. Across conditions such as heart failure, myocardial infarction, ischemia–reperfusion injury, atrial fibrillation, cardiotoxicity, sepsis-related cardiac injury, and cardiorenal syndrome, circRNAs appear to participate in context-dependent regulatory programs that link molecular perturbations to cellular and tissue-level outcomes [20,21,22,65,105].

Beyond their mechanistic relevance, circRNAs also show translational potential as circulating biomarkers and experimental therapeutic targets. Their stability, tissue-associated expression patterns, and detectability in plasma, serum, and extracellular vesicles support their use as candidate tools for diagnosis, prognosis, risk stratification, and disease monitoring [26,70,110]. Likewise, preclinical studies suggest that circRNA silencing, replacement, extracellular vesicle-mediated delivery, and regenerative circRNA-associated strategies may modulate disease-relevant pathways involved in fibrosis, hypertrophy, injury, cardiotoxicity, and cardiac repair [15,20,52,96].

However, the field remains at a transitional stage. Many circRNA candidates are still supported primarily by differential expression, predicted interactions, or isolated preclinical assays, and their functional relevance often remains incompletely validated. Key limitations include low abundance, inconsistent annotation, methodological heterogeneity, species differences, limited longitudinal evidence, uncertain cellular origin in biofluids, and major therapeutic barriers related to delivery, specificity, dosage control, immunogenicity, and long-term safety [10,16,26,69,82].

Future progress will require moving beyond descriptive circRNA profiling toward quantitative, cell-type-resolved, longitudinal, and functionally validated models of circRNA biology. Integrating human tissue studies, iPSC-derived cardiovascular models, perturbation-based functional genomics, multi-omics analysis, spatial approaches, and AI-guided network prioritization will be essential to distinguish circRNAs that are merely associated with disease from those that act as causal regulators or clinically informative biomarkers [40,64,69,109].

In conclusion, circRNAs represent an emerging regulatory layer linking molecular interactions with functional cardiovascular outcomes. Their relevance lies not only in their individual molecular targets, but in their capacity to coordinate multilayered regulatory programs involved in cardiac remodeling and disease progression. If current challenges in validation, standardization, reproducibility, and delivery are systematically addressed, circRNAs may contribute to future precision diagnostics and RNA-based therapeutic development in cardiovascular medicine.

## Figures and Tables

**Table 1:** Molecular mechanisms of circRNAs in cardiovascular biology.

Mechanism	Description	Regulatory Level	Representative circRNA	Biological Impact	Reference
miRNA sponge (ceRNA)	Sequestration of miRNAs via MREs, relieving mRNA repression	Post-transcriptional	circHRCR	Hypertrophy, fibrosis, apoptosis regulation	[14]
Protein scaffold/decoy	Interaction with RBPs, modulating protein complexes and signaling	Post-translational	circSMAD3, circITGa9	Fibrosis, cytoskeleton remodeling	[41,42]
Transcriptional regulation	Interaction with transcriptional machinery or chromatin regulators	Transcriptional	EIciRNAs	Gene expression modulation	[12]
Translation into peptides	Cap-independent translation via IRES or m6A	Translational	circCDYL, circNlgn	Hypertrophy, fibrosis	[18,19]
Mitochondrial regulation	Interaction with mitochondrial proteins affecting ROS and metabolism	Metabolic/Organelle	circSamd4, mecciRNAs	Oxidative stress, metabolic adaptation	[43,44]
Network-level integration	Coordination of multiple signaling pathways simultaneously	Systems-level	Multiple circRNAs	Remodeling, inflammation, regeneration	[40]

**Table 2:** Representative translated circRNAs involved in cardiovascular remodeling and disease through non-canonical translation mechanisms.

circRNA	Translation Initiation Mechanism	Encoded Peptide/Protein	Cardiovascular Context	Functional Role/Effect	Reference
circCDYL	m6A-dependent cap-independent translation	tCDYL-100	Cardiac hypertrophy	Promotes hypertrophic remodeling by disrupting the REST–CDYL–EHMT2 repression complex and activating pro-hypertrophic transcriptional programs	[18]
circNlgn	Cap-independent translation	Nlgn173	Cardiac fibrosis and remodeling	Promotes fibroblast proliferation and collagen deposition through LaminB1-associated nuclear signaling pathways	[19]
Circ_0036176 (circMYO9A)	IRES-dependent translation	Myo9a-208	Cardiac fibrosis	Suppresses cardiac fibroblast proliferation and may exert anti-fibrotic effects	[59]
circZNF609	IRES-mediated translation	circZNF609-derived peptide	Stress-responsive and muscle-associated remodeling contexts	Demonstrates functional circRNA translation capacity and supports the concept of circRNAs as dual-function regulatory molecules	[57]
General circRNA translation evidence	m6A- and IRES-mediated cap-independent translation	Multiple predicted peptides	Conceptual support for circRNA translation	Suggest potential proteomic diversification under ischemic, hypertrophic, and metabolic stress conditions	[13,57,58]

**Table 3:** Representative circRNAs in cardiovascular disease.

circRNA	Disease/Context	Main Mechanism	Targets (miRNA/Protein)	Functional Effect	Experimental Model	Reference
circHRCR	Cardiac hypertrophy	miRNA sponge	miR-223/ARC	Anti-hypertrophic	Mouse, cardiomyocytes	[14]
circCacna1c	Cardiac hypertrophy	miRNA sponge	miR-29b-2-5p/NFATc1	Pro-hypertrophic	In vitro, mouse	[47]
circSMAD4	Cardiac fibrosis	miRNA sponge	miR-671-5p/FGFR2	Promotes fibroblast activation	Mouse, fibroblasts	[20]
circHIPK3	Cardiac fibrosis	miRNA sponge	miR-29b-3p	Promotes ECM deposition	In vitro	[49]
circWhsc1	Myocardial injury/regeneration	Protein signaling modulation	TRIM59/STAT3/Cyclin B2	Enhances proliferation and repair	Mouse MI model, EV delivery	[15]
circMap4k2	Post-MI remodeling	miRNA sponge	miR-106a-3p/Azin1	Promotes regeneration, reduces fibrosis	Mouse (post-SVR)	[21]
circ_0001312	Cardiotoxicity	miRNA sponge	miR-409-3p/HMGB1	Reduces apoptosis and oxidative stress	In vitro, mouse	[52]
circITGa9	Cardiac fibrosis	Protein interaction	TPM3	Promotes cytoskeletal remodeling	Human tissue, in vitro	[41]
circSamd4	Mitochondrial regulation	Protein interaction	VCP	Reduces ROS, improves mitochondrial function	Mouse, cardiomyocytes	[44]

**Table 4:** Representative circRNA signatures with potential diagnostic, prognostic, or stratification relevance in cardiovascular disease.

circRNA	Cardiovascular Disease	Sample Source	Diagnostic/Prognostic Relevance	Potential Application	Validation	Reference
MICRA	Acute myocardial infarction (AMI)	Peripheral blood	Predicts left ventricular dysfunction after AMI	Post-MI risk stratification and prognosis	Multicenter human cohort	[87]
circRNA_081881	Heart failure	Plasma	Associated with HF severity and ventricular remodeling	Disease monitoring and progression assessment	Human cohort	[26]
circNFIX	Pressure overload-induced cardiac hypertrophy	Cardiac tissue/experimental models	Associated with miR-145-5p/ATF3-dependent hypertrophic remodeling	Molecular stratification of pressure overload-associated remodeling	Experimental validation	[48]
circHIPK3	Cardiac fibrosis and hypertrophy	Cardiac fibroblasts/experimental models	Correlates with fibroblast activation and adverse remodeling	Fibrosis burden assessment and therapeutic monitoring	Experimental with translational potential	[49]
circSMAD4	Cardiac fibrosis	Cardiac tissue	Reflects activation of profibrotic TGF-β-associated pathways	Fibrotic phenotype stratification	Experimental validation in vivo and in vitro	[20]
circCDYL	Pressure overload-induced hypertrophy	Cardiac tissue	Associated with pro-hypertrophic transcriptional remodeling	Molecular characterization of hypertrophic phenotypes	Functional mechanistic validation	[18]
circNlgn	Heart failure and fibrosis	Cardiac tissue	Linked to fibroblast proliferation and maladaptive remodeling	Identification of remodeling-associated molecular subtypes	Functional experimental validation	[19]
circWhsc1	Post-MI cardiac repair	Extracellular vesicles	Associated with regenerative signaling and cardiomyocyte proliferation	Experimental assessment of regenerative response and therapeutic stratification	Preclinical validation	[15]
circCacna1c	Cardiac hypertrophy	Cardiac tissue	Associated with NFAT-dependent hypertrophic signaling	Identification of stress-responsive hypertrophic remodeling	Experimental validation	[47]
circSNRK	Ischemic injury and cardiomyocyte survival	Cardiac tissue and experimental models	Associated with cardioprotective signaling and reduced apoptosis	Prediction of injury response and therapeutic responsiveness	Experimental validation	[51]

**Table 5:** Therapeutic strategies targeting circRNAs in cardiovascular disease.

Strategy	Intervention Type	Mechanism of Action	Example circRNA	Observed Effect	Model	Reference
ASO targeting (BSJ)	Antisense oligonucleotides	Degradation or inhibition of circRNA	circSMAD4	Reduced fibrosis	Mouse, fibroblasts	[20]
siRNA-mediated silencing	RNA interference	Knockdown of circRNA expression	circ_0001312	Reduced cardiotoxicity	Mouse, in vitro	[52]
Overexpression	Gene delivery (AAV/plasmid)	Restoration of protective circRNAs	circHRCR	Reduced hypertrophy	Mouse	[14]
Synthetic circRNAs	Engineered RNA molecules	miRNA sponging, protein modulation, or peptide coding	Synthetic circRNAs	Experimental modulation of gene networks	Experimental	[13]
EV-mediated delivery	Extracellular vesicles	Transfer of functional circRNAs between cells	circWhsc1	Enhanced regeneration	Mouse MI model	[15]
Biogenesis modulation	Targeting RBPs/ADAR	Regulation of circRNA production	QKI/ADAR pathways	Global circRNA remodeling	Experimental	[9]

## Data Availability

No new data were created or analyzed in this study. Data sharing is not applicable to this article.

## Abbreviations

The following abbreviations are used in this manuscript:

CVDs	Cardiovascular diseases
ncRNAs	Non-coding RNAs
circRNAs	Circular RNAs
MRE	MicroRNA response element
MBNL1	Muscleblind-like splicing regulator 1
QKI	Quaking RNA binding protein
A-to-I editing	Adenosine-to-inosine editing
ADAR	Adenosine deaminases acting on RNA
ecircRNA	Exonic circRNA
EIciRNA	Exon-intron circRNA
ciRNA	Circular intronic RNA
RBP	RNA-binding protein
IRES	Internal ribosome entry sites
m6A-dependent	N6-methyladenosine-dependent
ceRNA	Competing endogenous RNA
miRNA	microRNA
TPM3	Tropomyosin 3
VCP	Valosin-containing protein
mecciRNA	Mitochondrial circRNA
EV	Extracellular vesicle
